# Curcumin Ameliorates Age-Induced Tight Junction Impaired in Porcine Sertoli Cells by Inactivating the NLRP3 Inflammasome through the AMPK/SIRT3/SOD2/mtROS Signaling Pathway

**DOI:** 10.1155/2023/1708251

**Published:** 2023-02-17

**Authors:** Longfei Xiao, Zihao Fang, Qian Wang, Xihui Sheng, Xiaolong Qi, Kai Xing, Yong Guo, Hemin Ni, Xiangguo Wang, Yong Zhang

**Affiliations:** ^1^Animal Science and Technology College, Beijing University of Agriculture, Beijing, China; ^2^College of Veterinary Medicine, Gansu Agricultural University, Lanzhou, China

## Abstract

Blood-testis barrier (BTB) made of concomitant junction apparatus between Sertoli cells (SCs) is crucial for spermatogenesis. The tight junction (TJ) function is impaired in SCs with age, exhibiting an intimate relationship to testicular dysfunction induced by age. In this study, compared with those in young boars, TJ proteins (i.e., Occludin, ZO-1, and plus Claudin-11) were discovered to have reduced expressions in testes, and spermatogenesis ability declined in old boars. An *in vitro* age model for D-gal-treated porcine SCs was established, the performance of Curcumin as a natural antioxidant and anti-inflammatory compound in affecting the TJ function of SCs was appraised, and related molecular mechanisms were exploited. The results manifested that 40 g/L D-gal downregulated ZO-1, Claudin-11, and Occludin in terms of the expression in SCs, whereas Curcumin restored such expressions in D-gal-treated SCs. Using the AMPK and SIRT3 inhibiters demonstrated that activation of the AMPK/SIRT3 pathway was associated with Curcumin, which not only rescued the expression of ZO-1, Occludin, Claudin-11, and SOD2 but also inhibited the production of mtROS and ROS and the activation of NLRP3 inflammasome and release of IL-1*β* in D-gal-treated SCs. Furthermore, with mtROS scavenger (mito-TEMPO), NLRP3 inhibitor (MCC950) plus IL-1Ra treatment ameliorated D-gal-caused TJ protein decline in SCs. *In vivo* data also showed that Curcumin alleviated TJ impairment in murine testes, improved D-gal-triggered spermatogenesis ability, and inactivated the NLRP3 inflammasome by virtue of the AMPK/SIRT3/mtROS/SOD2 signal transduction pathway. Given the above findings, a novel mechanism where Curcumin modulates BTB function to improve spermatogenesis ability in age-related male reproductive disorder is characterized.

## 1. Introduction

Aging is referred to a degradation process that is generalized, multiaspect, complicated, and progressive under the influences of multiple factors including genetics, age, environments, and lifestyles [1]. It has an impact on the reproductive ability of individuals. Among the male reproductive organs, testes are capable of generating sperm to sustain the male reproductive function and secreting androgen for the purpose of regulating spermatogenesis modulation and male sexual function maintenance [2]. The animal model of senescence established showed associations of aging or agents utilized with the reduction in the reproductive dysfunction, steroidogenesis, spermatogenesis, and sexual dysfunction of males, causing testis impairment or decaying of the testis [3]. As the unique somatic cells in the seminiferous tubules, Sertoli cells (SCs) are directly connected with spermatogenic cells, and they can produce a blood-testis barrier (BTB) with adjacent SCs in the tight junction (TJ), thereby enabling the germ cells to develop in a special protective environment [4].TJ is an important part of BTB, and the related proteins play a crucial role in maintaining the integrity and normal opening and closing of BTB [5]. Recent studies have shown that aging can lead to decreased function of SCs and damage to TJ structures, resulting in testicular spermatogenesis dysfunction [6]. In general, the reproductive ability of breeding boars starts after sexual maturity, reaches the peak at 2-3 years old, and then declines year by year [7]. Therefore, restoring and improving the TJ function of SCs are crucial to prevent male testicular spermatogenesis dysfunction due to aging.

The associations of aging with increased inflammation and chronic diseases have now been well established. In terms of inflammaging, the elevated levels of inflammatory cytokines in old people are considered as the driver of decreased success in aging and health span [8, 9]. The inflammation associated with age in several organs is likely to cause functional decline even without a defined disease. As demonstrated by the latest studies, the NOD-like receptor family pyrin domain containing 3 (NLRP3) inflammasome becomes an important member of the leading regulators for inflammation and metabolic disturbance related to age [10]. The NLRP3 inflammasome monomer, a multisubunit protein, can not only activate Caspase-1 but also induce the delivery of interleukin-1*β* (IL-1*β*) and IL-18. These canonical cytokines mediate inflammatory responses in many kinds of cells [11, 12], so they could cause or exacerbate the cell or organ inflammation contributing to the development of diseases.

As a Ser/Thr kinase with a widespread expression, AMP-activated protein kinase (AMPK) is an energy sensor able to supervise the AMP/ATP level, which can control cellular metabolism by means of recovering ATP [13]. Besides the pivotal function of modulating the dynamics of cellular energy, the AMPK signaling pathway participates in the control of inflammation and oxidative stress [14]. It has been reported in research that oxidative stress is restrained, and mitochondrial DNA integrity is enhanced *via* Sirtuin 3 (SIRT3) after the activation of the AMPK signaling pathway [15, 16]. With mitochondrial reactive oxygen species (mtROS) as the representative, ROS serve as one category of critical factors stimulating the activation of NLRP3 inflammasome [17]. SIRT3, one of the SIRT family, is mainly capable of modulating mtROS levels through changing the acetylation of such leading mitochondrial antioxidant enzymes as isocitrate dehydrogenase 2, manganese superoxide dismutase 2 (SOD2), plus glutathione peroxidase [18]. It is more crucial that SOD2 activity can be strengthened by SIRT3 *via* direct conjugation with and deacetylation of SOD2, subsequently significantly affecting mtROS homeostasis together with the activation of NLRP3 inflammasome [19].

Curcumin (chemical name: 1, 7-bis (4-hydroxy-3-methoxyphenyl)-1, 6-heptadiene-3, 5-dione) has been proven to possess the antioxidant [20], anti-inflammatory [21], antiapoptotic [22], and antibacterial [23] functions by scientific research since it is recognized as an active ingredient of turmeric rhizomes (alias *Curcuma longa Linn*). A number of clinical studies conducted over the past few years uncovered that Curcumin exerts a therapeutic effect on numerous chronic disorders (psychological, pulmonary, neurological, cardiovascular, neoplastic, metabolic diseases, etc.) [24]. It has been demonstrated that Curcumin maintains the integrity of TJ proteins to protect the intestinal [25], blood-brain [26], and alveolar epithelial barriers [27]. However, the precise contribution of Curcumin-mediated protection remains to be elucidated against aging-triggered male reproductive dysfunction.

Given that the intervention of the TJ function in SCs may be implicated in the aggravation of spermatogenesis ability dysfunction, our present study aims to examine the effects of Curcumin on D-gal-mediated aging-induced TJ dysfunction in porcine SCs in culture and to explore the underlying molecular events. Additionally, we validated the association of the Curcumin modulation of BTB with the spermatogenesis ability in a model of D-gal induced age mouse subject to a chronic administration of Curcumin. As a result, our findings reveal a novel molecular mechanism of Curcumin as a potentially protective agent against the aging-triggered male reproductive dysfunction.

## 2. Materials and Methods

### 2.1. Porcine Testis Collection

Sperm were collected from 10 (12-18 months old) young and 6 (5-6 years old) old Landrace boars in the local station within 3 days before slaughter. Indicators related to the sperm quality (density, motility, and deformity rate) were detected with the WLJX-9000 Weili Color Sperm Analysis System, a computer-aided sperm analysis system (CASA system; Weili New Century Science & Tech Development Co., Ltd., Beijing, China), where the confidence interval of sperm quality was calculated as the standard value. Three young porcine testes were randomly collected from those with the standard value, and three old porcine testes with remarkably lower sperm quality than the standard value calculated from the young ones selected. A sample with a size of about 3 × 3 cm was collected from the middle part of testis, which was quickly refrigerated using liquid N_2_ and then preserved in a −80°C environment for acquisition of RNA and protein. Fixation with neutral paraformaldehyde phosphate buffer (4%, pH 7.4) was performed for the remaining part for subsequent hematoxylin-eosin (HE) staining and immunofluorescence analysis.

### 2.2. Cell Culture

Normal Landrace boars aged 7-15 days old were chosen to acquire the testicular tissues. After rinsing the tissues by phosphate buffer saline (PBS) containing penicillin (100 IU/mL) and streptomycin (100 mg/mL) for 3 times, the separated SCs were subjected to culture *as per* a previous method which was mildly modified [28]. Next, the tunica albuginea of every testis was removed following rinsing with the culture medium in a sterile environment. Then, the testicular parenchyma was obtained, chopped, and digested with collagenase type IV (1 mg/mL, 15 min, and 37°C). Moreover, the convoluted seminiferous tubules were collected under a stereo microscope after 3 times of PBS washing, followed by 30 min of digestion of the tissues using collagenase type IV. Afterward, cells were centrifuged for 5 min at 1, 000 g/min and collected and rinsed with the culture medium for 3 times. Besides, DMEM-F12 containing 10% FBS (Hyclone Laboratory) plus antibiotics (penicillin G at 50 IU/mL together with streptomycin at 50 *μ*g/mL) was utilized to resuspend the cell pellets in an incubator under a humidified atmosphere (34°C, 95% air +5% CO_2_). Being incubated for 72 h, the cells were subjected to 20 mM hypotonic Tris-HCl solution (pH 7.4) processing, followed by 2 min of gentle shaking to eliminate residual germ cells. After that, the Tris-HCl solution was abandoned. The cells were inoculated into 6-well plates (nearly 1.5 × 10^5^ cells/well) when the confluence reached 80%, which were left in a 34°C humidified incubator (95% O_2_+5% CO_2_, 24 h).

Immunofluorescence analysis was conducted on the cells collected after culture. Briefly, SCs underwent 3 times of washing in PBS, 15 min of room-temperature fixation with 4% paraformaldehyde, and washing for 3 times again. Subsequently, the blocking buffer composed of 5% BSA and 0.1% Triton X-100 in PBS, the primary rabbit anti-SHBG polyclonal antibody (diluted at 1 : 100; Bioss, Beijing, China; bs-1175R), and the anti-SOX9 mouse monoclonal antibody (diluted at 1 : 100; Proteintech, Wuhan, China; 67439-1-lg) were used for overnight incubation of the cells at 4°C. Later, the cells were washed 3 times in PBS, cultured for 45 min using FITC-conjugated goat anti-rabbit IgG (H + L) antibody (dilution rate 1 : 200; TransGen, Beijing, China) plus PE-conjugated goat anti-mouse IgG (H + L) antibody (dilution rate 1 : 200; TransGen, Beijing, China), and counterstained by Hoechst33342 (Beyotime, Nanjing, China) for nucleus identification.

Next, the serum-free DMEM/F12 was used for 12 h of cell culture prior to experiments, and then D-gal (at 5, 10, 20, or 40 g/L in final concentration to induce aging, Med Chem Express) and/or Curcumin (5, 10, and 20 *μ*M, Med Chem Express, New Jersey, USA) were/was added to treat the cells for 48 h. Regarding the research on inhibition, the cells underwent incubation with or without compound C (AMPK inhibitor, at 10 *μ*M, Med Chem Express), 3-TYP (SIRT3 inhibitor, at 50 *μ*M, Med Chem Express), mito-TEMPO (mtROS scavenger, at 50 *μ*M, Med Chem Express), MCC950 (NLRP3 inhibitor, at 10 *μ*M, Med Chem Express), and/or IL-1 receptor antagonist (IL-1Ra, at 20 ng/mL, Med Chem Express) for 2 h before treatment.

### 2.3. Animals and Treatment

Relevant regulations issued by the China Council on Animal Care were selected as the standards for processing all the experimental animals. The guidelines formulated by the Animal Ethics Committee of Beijing University of Agriculture (Permit No.: SYXK(JING)2021-0001) were set as the criteria for all the procedures.

C57BL/6 male mice provided by the Animal Core Facility of Beijing Vital River Laboratory (Beijing, China), 50 in total, with an age of 7–8 weeks old, were selected and bred in an animal house with a 12 h dark/light cycle, in which the temperature and humidity were controlled at 20–25°C and 40–70%, respectively. Moreover, the mice were offered with food and water *ad libitum* in the whole process of the study. Following 1 week of acclimatization, the mice were randomly assigned into 5 groups (*n* = 10), namely, control group, Curcumin group (100 mg/kg, 200 mg/kg, and 400 mg/kg), and D-gal group. In the D-gal group, the mice were daily administered with D-gal (200 mg/kg/day) through subcutaneous injection for 60 days following a previous method [29], while the mice in the control group received 60 days of saline injection in an equal volume. As for the Curcumin group, Curcumin at 100, 200, and 400 mg/kg/day was administrated *via* gavage following the daily D-gal injection for 60 days.

All the mice were sacrificed under general anesthesia on the last day of drug application (i.e., day 60), during which pentobarbital sodium (150 mg/kg) was intraperitoneally injected for anesthesia induction. After resection, the left testis was rapidly preserved at -80°C for bioassays, whereas the right testis was subjected to 4% paraformaldehyde fixation for histological experiments and immunofluorescence analysis.

### 2.4. Analysis of Murine Sperm

The cauda epididymis was harvested and extruded to obtain the sperm, which were suspended using Modified HTF Medium (In Vitro Care, MD, USA) mixed with 10% FBS. After 5 min of 37°C incubation, the motility and deformity rate of all the sperm samples in a single volume of 10 *μ*L were analyzed by virtue of the WLJX-9000 Weili Color Sperm Analysis System (CASA system; Weili New Century Science & Tech Development Co., Ltd., Beijing, China).

### 2.5. Histological Analysis

The porcine and murine testicular tissues embedded in paraffin were made into 4 *μ*m thick sections for HE staining. The Olympus-DP73 optical microscope (Tokyo, Japan) was employed to assess the dynamic changes in the histology of the testes.

### 2.6. Immunofluorescence Analysis

First, the anti-SOX9 mouse monoclonal antibody (a specific SC marker) together with the rabbit polyclonal antibody against ZO-1 (diluted at 1 : 100; bs-1329R; Bioss), Claudin-11 (dilution rate 1 : 100; bs-21509R; Bioss), or Occludin (diluted at 1 : 100; bs-10011R; Bioss) was applied to incubate the testicular tissue sections (4 *μ*m) at 4°C overnight. Subsequently, the tissue sections were cleaned by PBS for 3 times, and the goat anti-rabbit IgG (H + L) antibody conjugated with FITC (diluted at 1 : 200; TransGen, Beijing, China) and the goat anti-mouse IgG (H + L) antibody labeled with PE (dilution rate 1 : 200; TransGen, Beijing, China) were utilized for 45 min of incubation. Finally, Hoechst 33342 was adopted to stain the cell nuclei *as per* the methods described before [28]. A fluorescence microscope (Nikon, Tokyo, Japan) was employed to observe the protein expression and colocalization.

### 2.7. Western Blotting

After washing in ice-cold PBS, the acquired tissues and cells were processed with ice-cold radioimmunoprecipitation assay (RIPA) lysis buffer complemented with phenylmethylsulfonyl fluoride (PMSF, at 1 mM), followed by Western blotting in accordance with the previously mentioned methods [28], with Occludin (diluted at 1 : 1000; bs-10011R; Bioss), ZO-1 (dilution rate 1 : 1000; bs-1329R; Bioss), p-AMPK (Ser485) (diluted at 1 : 1000; #2537; Cell Signaling Technology; Danvers; USA), Claudin-11 (dilution rate 1 : 1000; bs-21509R; Bioss), AMPK (Ser485) (1 : 1000 dilution; #2532; Cell Signaling Technology), SIRT3 (diluted at 1 : 1000; 10099-1-AP; Proteintech), *β*-actin (1 : 3,000 dilution; bs-0061R; Bioss), SOD2 (dilution rate 1 : 1000; bs-23402R; Bioss), NLRP3 (1 : 1000 dilution; 19771-1-AP; Proteintech), and IL-1*β* (dilution rate 1 : 1000; #12703; Cell Signaling Technology) as the primary antibodies, as well as the HRP-labeled goat anti-rabbit antibody (diluted at 1 : 3000, bs-0295-HRP; Bioss) as the secondary antibody. Ultimately, the ECL solution was applied to measure the bands, and the Image J software was employed to determine the signals.

### 2.8. ROS Assessment

DCFH-DA provided by Beyotime Institute of Biotechnology (Jiangsu, China) or MitoSOX™ Red purchased from Invitrogen (Carlsbad, CA, USA) was used to detect the levels of total ROS and mtROS in line with the manufacturer's instructions. First of all, a 96-well microplate was seeded with SCs (density: 5000 cells/well) that were subsequently handled according to the instructions. Next, 10 *μ*mol/L DCFH-DA or 5 *μ*mol/L MitoSOX reagents was added to the cells in the dark for 30 min of incubation at 37°C, which were gently cleaned with PBS three times. The Gemini XPS Microplate Reader (Molecular Devices, Gothenburg, Sweden) was used to examine the fluorescence intensity of total ROS and mtROS. At last, the Hoechst 33342-stained cell nuclei were subjected to microscopic analyses under a fluorescence microscope (Nikon, Tokyo, Japan).

### 2.9. Testis Oxidative Stress Evaluation

The activities of SOD and CAT (enzymatic antioxidants) and the level of MDA as a lipid peroxidation marker were measured to evaluate the oxidative stress on porcine and murine testis *as per* the instructions of MDA (A003-1), SOD (A001-1), and CAT (A007-1) kits (Jiancheng Bioengineering Institute, Nanjing, China) that were commercially available.

### 2.10. Measurement of IL-1*β*

With the culture medium as the subject, enzyme-linked immunosorbent assay (ELISA) was carried out to detect IL-1*β* concentration, of which the specific procedures were presented in Porcine IL-1*β* ELISA Kit (JL21874) bought from Jianglaibio (Shanghai, China). Each sample was measured twice for the absorbance at 450 nm, with the values of negative control (blanks without any sample) removed. The minimum detectable concentration of IL-1*β* (in pg/mL) was 1 pg/mL.

### 2.11. Data Analysis

SPSS Version 19.0 (SPSS Inc., Chicago, IL, USA) was employed for statistical analyses. The normality and homoscedasticity of all the data were examined, and one-way analysis of variance plus Duncan's Multiple Range Test was subsequently implemented to determine the significant differences in data. The mean ± SEM was adopted to present the quantitative data. *P* < 0.05 denoted that the difference was statistically significant.

## 3. Results

### 3.1. D-Gal-Induced Aging Led to Testicular Dysfunction in Boars

**Figure 1:**
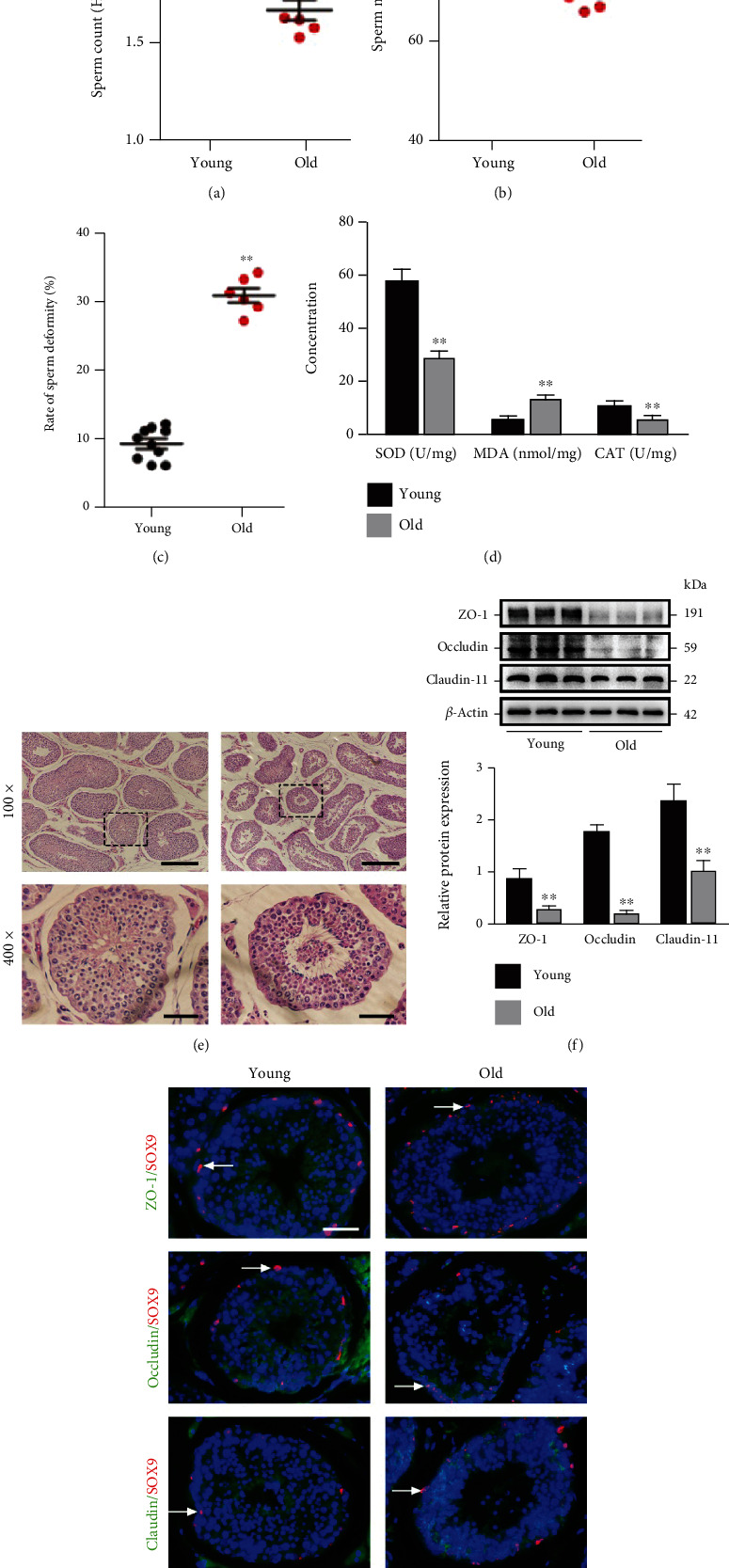
D-gal-induced aging led to testicular dysfunction in boars. The sperm content (a), motility (b), and deformity rate (c) of young and old boars are detected. (d) SOD, MDA, and CAT content in young and old porcine testes is detected. (e) The histological variations in the testes are appraised with HE staining. The changes in seminiferous tubules (original magnification 100×, scale bar = 200 *μ*m) are presented in the upper panels. The magnified images of the boxed areas are exhibited in the lower panels (original magnification 400×, scale bar = 50 *μ*m). (f) The relative expressions of Claudin-11, ZO-1, and Occludin proteins in testicular tissues are determined with Western blotting. The arrow indicates SCs. (g) Representative images of immunofluorescence for colocalizing ZO-1, Occludin, and Claudin-11 (green) by virtue of SOX9 (red) in the testes are presented. *β*-Actin was used for the loading control. The mean ± SEM was used for value presentation; ^∗∗^*P* < 0.01 vs. the young group.

The sperm quality in the young and old boars was assessed first. As shown in Figures 1(a)–1(c), the old boars had evidently lower sperm content and motility as well as a higher deformity rate than the young ones (*P* < 0.01). Moreover, in contrast to those in the young porcine testes, the content of SOD and CAT declined significantly (*P* < 0.01), but the MDA content rose prominently in the old ones (Figure 1(d), *P* < 0.01). Evaluation of testis histological sections displayed normal histological appearance and seminiferous tubule, demonstrating a normal spermatogenesis process in the young control group, while the testicular tissues from the aged group were found with severely atrophied seminiferous tubules. Due to the spermatogenic cells lost in the tubules, the arrangement of spermatogenic cells was disordered, and the numbers of sperm were decreased in the lumen (Figure 1(e)).

Given that Claudin-11, ZO-1, and Occludin serve as the leading proteins implicated in TJ formation, Western blotting and immunofluorescence analysis were conducted to measure such protein expression levels. The results indicated that Claudin-11, ZO-1, and Occludin displayed significantly decreased relative protein expressions in the old testicular tissues compared to the young ones (Figures 1(f) and 1(g)). In summary, the spermatogenesis and TJ function of the testes are injured in old boars according to the aforementioned data.

### 3.2. Curcumin Inhibited D-Gal-Induced Decreased Barrier Function in SCs In Vitro

**Figure 2:**
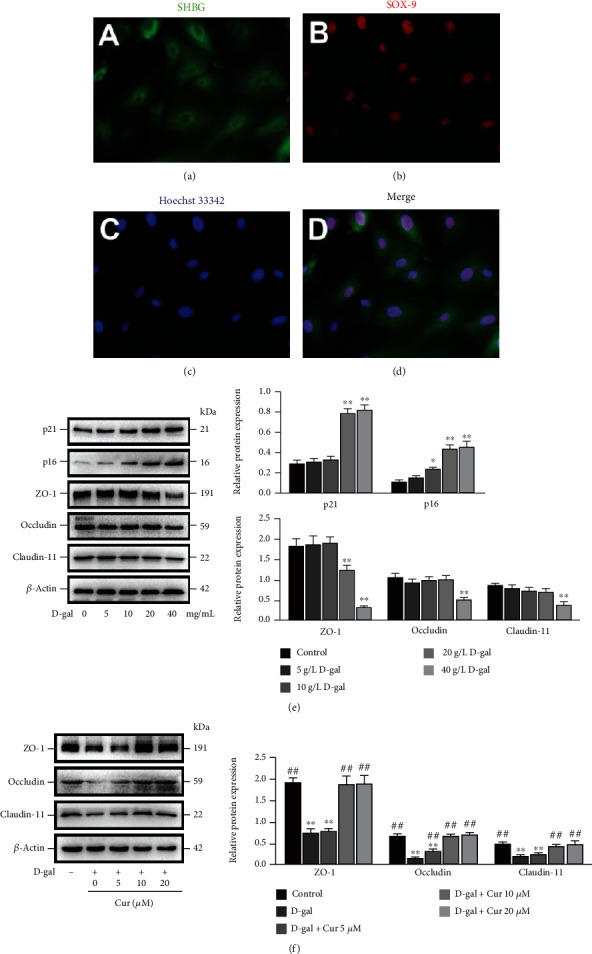
Curcumin inhibits D-gal-induced decreased barrier function in SCs *in vitro*. (a–d) SCs with immunofluorescence of SHBG (green) and SOX9 (red) are identified. (e) SCs are treated with 0, 5, 10, 20, and 40 g/L D-gal for 48 h. Western blotting of Claudin-11, p21, ZO-1, p16, and Occludin proteins. (f) SCs are subjected to 48 h of treatment with D-gal (40 g/L) and/or Curcumin at the concentrations required. Western blotting was conducted to analyze ZO-1, Occludin, and Claudin-11 proteins, with *β*-actin as the loading control. The mean ± SEM is applied to exhibit the values; ^∗^*P* < 0.05 and ^∗∗^*P* < 0.01 vs. the control group; ^#^*P* < 0.05 and ^##^*P* < 0.01 vs. the D-gal treatment group.

For the purpose of validating whether aging affects the BTB disruption by means of its influence on SCs, the porcine SCs were primarily cultured. The porcine SCs manifested a round or square shape, their cytoplasm was green in the staining with anti-SHBG antibody, and the nuclei were red under the anti-SOX9 antibody staining (Figures 2(a)–2(d)). The premature aging stimulated through chronic exposure to D-gal has similar characteristics to natural aging [2]. Therefore, the D-gal-treated SCs were selected to mimic aging in the present study. After 48 h of processing of the SCs with D-gal at varying concentrations (0, 5, 10, 20, and 40 g/L) (Figure 2(e)), the cells cultured using D-gal doses caused continuous rises in the expressions of aging-associated proteins p16 and p21, indicating that D-gal treatment can mimic the aging of SCs. Moreover, 0-20 g/L D-gal had no significant effect on TJ protein expressions (Claudin-11, ZO-1, and Occludin), while 40 g/L D-gal could notably lower these protein expressions in comparison to the control group (*P* < 0.01).

Second, the role of Curcumin in influencing the D-gal-induced decrease in barrier function in SCs was investigated. Curcumin dose-dependently diminished the Claudin-11, ZO-1, and Occludin protein expressions in the 40 g/L D-gal-stimulated SCs (Figure 2(f)). To sum up, D-gal can trigger the aging of SCs and disrupt TJ, while Curcumin can inhibit the decreased barrier function in aged SCs *in vitro*.

### 3.3. Activation with AMPK/SIRT3 Signaling Was Associated with the Function of Curcumin in Inhibiting D-Gal-Induced Decreased Barrier in SCs In Vitro

**Figure 3:**
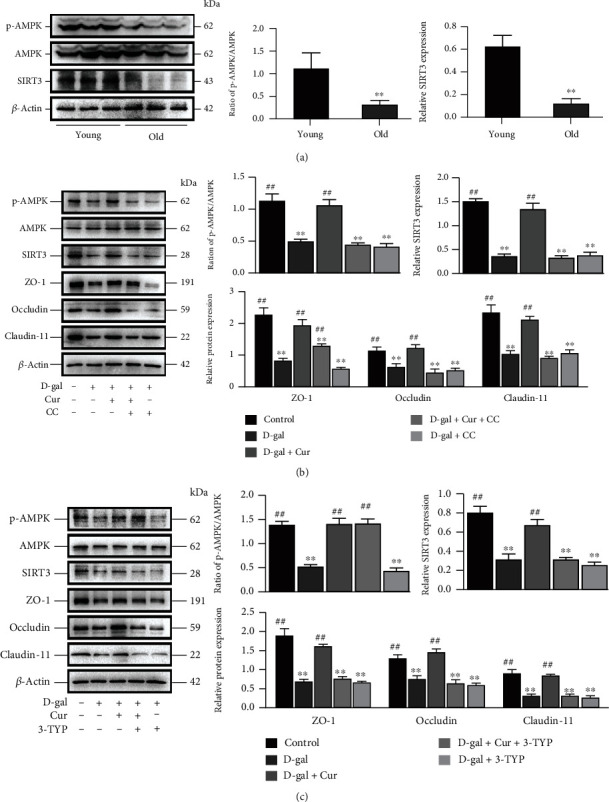
Activation with AMPK/SIRT3 signaling is associated with the function of Curcumin in inhibiting D-gal-induced decreased barrier function in SCs *in vitro*. (a) p-AMPK, SIRT3, and AMPK in porcine tissues are analyzed using Western blotting. (b) SCs are treated with D-gal (40 g/L), compound C (10 *μ*M), and/or Curcumin (10 *μ*M) for 48 h. p-AMPK, AMPK, SIRT3, and TJ proteins are researched by means of Western blotting. (c) SCs are treated for 48 h with D-gal (40 g/L), 3-TYP (50 *μ*M), and/or Curcumin (10 *μ*M). p-AMPK, AMPK, SIRT3, and TJ proteins are examined through Western blotting, with *β*-actin as the loading control. The mean ± SEM is used to present the values; ^∗∗^*P* < 0.01 vs. the control group; ^##^*P* < 0.01 vs. the D-gal treatment group.

Subsequently, the molecular mechanisms in which Curcumin inhibits D-gal-induced decreased barrier function in SCs were explored. AMPK and SIRT3 have shown a key role during aging [14]. The AMPK phosphorylation and the SIRT3 expression were discovered to be apparently lower in the old porcine testis than those in the young one (Figure 3(a), *P* < 0.01). Moreover, 10 *μ*M Curcumin increased p-AMPK and SIRT3 expressions in SCs processed with D-gal. Combined with compound C (AMPK inhibitor) and 3-TYP (SIRT3 inhibitor), however, it was revealed that the repression of AMPK and SIRT3 lowered ZO-1, Occludin, and Claudin-11 protein expressions in D-gal-treated SCs (Figures 3(b) and 3(c)). Furthermore, compound C inhibited the stimulating effect of Curcumin on p-AMPK and SIRT3 expressions in D-gal-induced SCs, while 3-TYP only inhibited SIRT3 expression but not p-AMPK expression (Figures 3(b) and 3(c)). In general, the above findings imply that the role of Curcumin in inhibiting D-gal-induced decreased barrier function in SCs is depended on the activation with the AMPK/SIRT3 signaling pathway.

### 3.4. Curcumin Inhibited D-Gal-Induced Decreased Barrier Function via Suppressing ROS and mtROS Accumulation in SCs In Vitro

**Figure 4:**
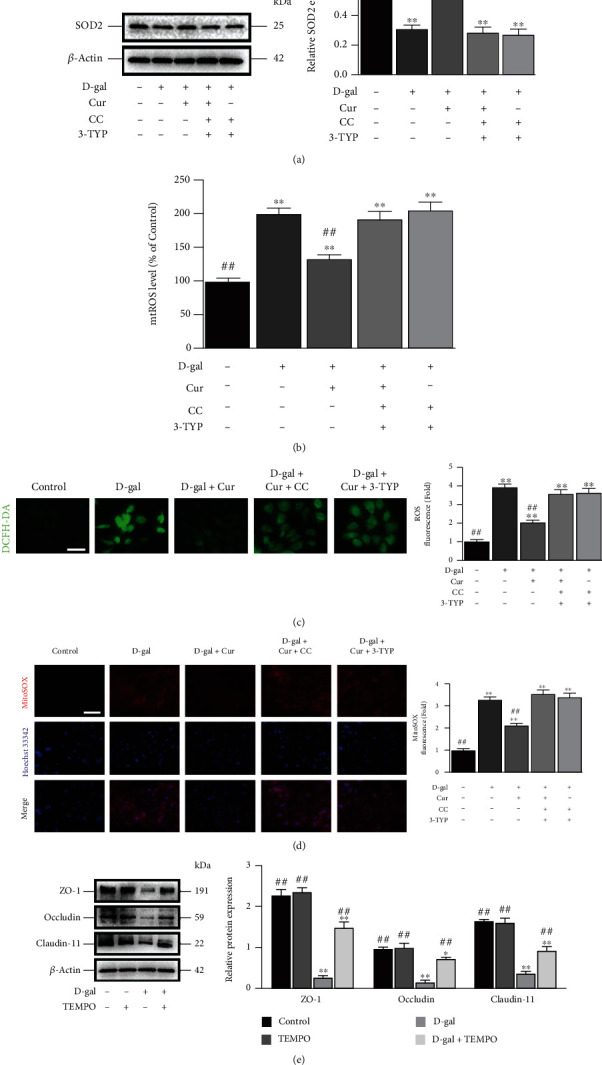
Curcumin inhibits D-gal-induced decreased barrier function via inhibiting ROS and mtROS accumulation in SCs *in vitro*. (a) SCs undergo 48 h of treatment with D-gal (40 g/L), compound C (10 *μ*M), 3-TYP (50 *μ*M), and/or Curcumin (10 *μ*M). Western blotting is performed for SOD2 proteins. (b) The mtROS levels are measured using MitoSOX™ Red. (c) Total ROS levels are detected with DCFH-DA (scale bar = 50 *μ*m). (d) The mtROS levels are quantified by virtue of a fluorescence spectrometer (scale bar = 50 *μ*m). (e) SCs are treated with D-gal (40 g/L) and/or mito-TEMPO (50 *μ*M) for 48 h. ZO-1, Occludin, and Claudin-11 proteins are probed into *via* Western blotting, for which *β*-actin is determined as the loading control. The values are expressed in the format of mean ± SEM; ^∗∗^*P* < 0.01 vs. the control group; ^##^*P* < 0.01 vs. the D-gal treatment group.

Previous studies have identified that ROS and mtROS can cause barrier function disorders [30]. As an important mtROS scavenger [18, 19], SOD2 in D-gal-treated SCs was monitored for its activity. Curcumin was able to elevate SOD2 expression and lower the levels of total ROS and mtROS in D-gal-treated SCs, while combined with compound C and 3-TYP, the stimulating effect of curcumin on SOD2 expression and the inhibition effect on total ROS and mtROS production were inhibited in D-gal-treated SCs (Figures 4(a)–4(d)). Furthermore, mito-TEMPO (a scavenger specific to mtROS) preprocessing increased the protein expressions of ZO-1, Claudin-11, and Occludin in D-gal-treated SCs (Figure 4(e)). These data validated the inhibitory effects on the production of total ROS and mtROS involved in the function of Curcumin in repressing the D-gal-induced decreased barrier function process.

### 3.5. Curcumin Restrained D-Gal-Induced Decreased Barrier Function via Inhibiting the Activation of NLRP3 Inflammasome and the Delivery of IL-1*β* in SCs In Vitro

**Figure 5:**
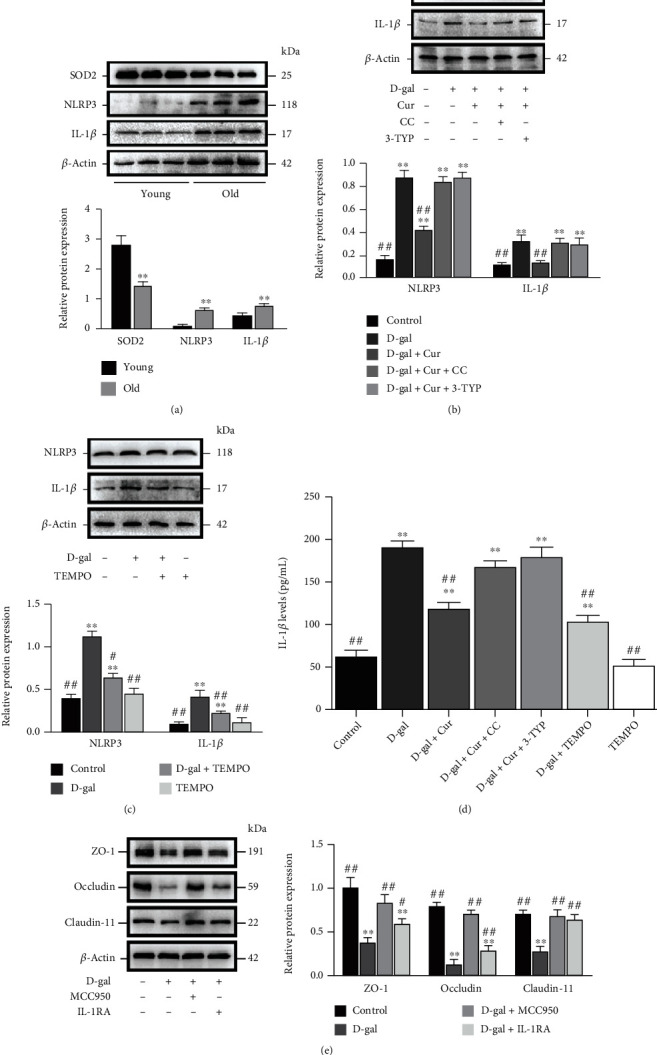
Curcumin inhibits D-gal-induced decreased barrier function *via* inhibiting activated NLRP3 inflammasome and released IL-1*β* in SCs *in vitro*. (a) Western blotting is carried out to analyze SOD2, NLRP3, and IL-1*β* in porcine tissues. (b) SCs are treated for 48 h with D-gal (40 g/L), compound C (10 *μ*M), 3-TYP (50 *μ*M), and/or Curcumin (10 *μ*M). NLRP3 and IL-1*β* proteins are explored through Western blotting. (c) SCs are treated with D-gal (40 g/L) and/or mito-TEMPO (50 *μ*M) for 48 h to analyze NLRP3 and IL-1*β* proteins using Western blotting. (d) IL-1*β* secretion is investigated by ELISA analysis using cell culture supernatant fractions. (e) SCs receive 48 h of treatment under D-gal (40 g/L), MCC950 (10 *μ*M), and/or IL-1Ra (20 ng/mL), followed by analysis of ZO-1, Occludin, and Claudin-11 proteins by virtue of Western blotting, with *β*-actin as the loading control. The mean ± SEM is used as the expression format for values; ^∗^*P* < 0.05 and ^∗∗^*P* < 0.01 vs. the control group; ^#^*P* < 0.05 and ^##^*P* < 0.01 vs. the D-gal treatment group.

mtROS is a pivotal player in stimulating NLRP3 inflammasome activation, and the activation of NLRP3 inflammasome could also cause barrier function disorder. The old porcine testes exhibited an obviously decreased SOD2 expression and significantly increased NLRP3 and IL-1*β* protein expression levels compared with the young ones (Figure 5(a)). Moreover, Curcumin and mito-TEMPO significantly decreased NLRP3 and IL-1*β* in terms of the protein expressions, as well as IL-1*β* release, in D-gal-treated SCs, while the results combined with compound C as the AMPK inhibitor and 3-TYP as the SIRT3 inhibitor showed that the inhibition effect of Curcumin was repressed (Figures 5(b)–5(d)).

Furthermore, to evaluate whether the barrier function disorder is attributed to D-gal-triggered NLRP3 activation and IL-1*β* release increase, MCC950 (a selective NLRP3 inhibitor) and IL-1Ra were used. After the application of 10 *μ*M MCC950 and 20 ng/mL IL-1Ra, the decreased Claudin-11, Occludin, and ZO-1 protein expressions were partly inhibited in D-gal-treated SCs (Figure 5(e), *P* < 0.01). In summary, the inhibition of the activation of NLRP3 inflammasome together with the dissipation of IL-1*β* is also involved in the function of Curcumin in the inhibiting process of D-gal-induced decreased barrier function.

### 3.6. Curcumin Restrained BTB Disruption by Means of the Inactivation of NLRP3 Inflammasome via the SIRT3/AMPT/SOD2 Signaling Pathway in Murine Testes Injected with D-Gal

**Figure 6:**
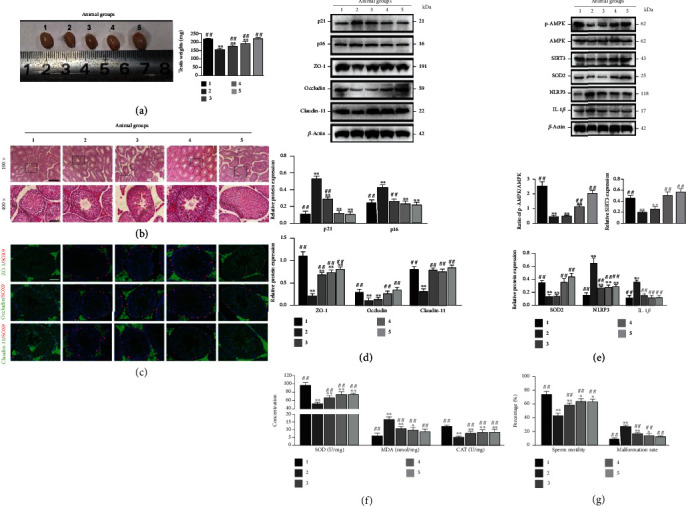
Curcumin inhibits BTB disruption through inactivating the NLRP3 inflammasome *via* the SIRT3/AMPT/SOD2 signaling pathway in D-gal-treated murine testes. The mice were allocated into control group (group 1, no D-gal, without treatment), model group (group 2, with D-gal, without treatment), Curcumin-treated group (group 3, with 100 mg/kg Curcumin + D-gal), curcumin-treated group (group 4, with 200 mg/kg Curcumin + D-gal), and Curcumin-treated group (group 5, with 400 mg/kg Curcumin + D-gal). (a) Testicular weight change in the mice is detected. (b) The histological changes in testes were appraised with HE staining. The upper and lower panels manifest the changes in seminiferous tubules (original magnification 100×) and the magnified images of the boxed areas (original magnification 400×), respectively. (c) Typical immunofluorescence images for colocalizing ZO-1, Occludin, and Claudin-11 (green) by virtue of SOX9 (red) in the testes are displayed. (d) Western blotting is carried out to exploit ZO-1, Occludin, and Claudin-11 proteins. (e) p-AMPK, AMPK, SIRT3, SOD2, NLRP3, and IL-1*β* proteins are examined using Western blotting. (f) Changes in SOD, MDA, and CAT content are examined. (g) The changes in sperm motility and deformity rate are measured. *β*-Actin is applied as the loading control, and the format of values is mean ± SEM; ^∗^*P* < 0.05, ^∗∗^*P* < 0.01 vs. the control group; ^#^*P* < 0.05 and ^##^*P* < 0.01 vs. the D-gal treatment group.

Finally, the roles of Curcumin in impacting BTB disruption in murine testes chronically injected with D-gal were evaluated. As shown in Figure 6(a) , D-gal was capable of suppressing testis development and significantly decreasing the weight of the testis, while Curcumin stimulated testis development and increased the weights of testis in mice chronically injected with D-gal. Moreover, the results of HE staining for examining the histology revealed that the testicular tubules had markedly restored morphology and structure, and the sperm cells exhibited a raised number of layers after Curcumin treatment in mice chronically injected with D-gal (Figure 6(b)). Immunofluorescence staining and Western blotting for Claudin-11, Occludin, and ZO-1 demonstrated that the expressions of these TJ proteins were decreased but induced by Curcumin treatments in the aged model of testes (Figures 6(c) and 6(d)). Furthermore, 200 and 400 mg/kg Curcumin treatment significantly increased the protein abundance of p-AMPK, SIRT3, and SOD2, while decreased the expression of NLRP3 and IL-1*β* proteins in the testes of the aged group (Figure 6(e)), which were consistent with the results obtained in D-gal-treated SCs. The SOD and CAT content and sperm motility in testes were also increased by Curcumin treatment, while the MDA content and deformity rate were greatly decreased in mice chronically injected with D-gal (Figures 6(f) and 6(g), *P* < 0.01). To sum up, it was testified by the *in vivo* data that Curcumin ameliorates spermatogenesis and decreases BTB impairment in testes through inactivating the NLRP3 inflammasome *via* the SIRT3/AMPK/SOD2 signaling pathway in D-gal-caused aged mice, at least in part through contributing to recovering TJ function in SCs.

## 4. Discussion

As the age increases, impairment or decaying of the testicular structure and function occurs, thus disrupting the male reproductive function plus the spermatozoa count and quality [31]. The SCs enclosing the germ cells under development in the process of spermatogenesis serve as the orchestrators influencing the development and spermatogenesis of germ cells due to the function of nutrient provision and participation in BTB formation [3, 4]. It has been demonstrated that aging is able to downregulate TJ proteins in terms of the expression and localization, so as to impair the BTB and worsen the destruction of the seminiferous epithelium [6], ultimately inducing spermatogenic dysfunction or even infertility. According to the results in this study, Claudin-11, ZO-1, and Occludin had evidently decreased expression levels in the old porcine testes compared with those in the young ones, implying that aging is probably a vital factor for BTB damage and reproductive capacity reduction in boars.

For the purpose of prolonging the useful life of breeding boars, it is essential to understand the aging mechanism of SCs considering their critical functions in spermatogenesis and construction of BTB integrity, together with the unfavorable effects of aging on SC functions. As revealed by this study, the progress of aging was simulated using SCs through 48 h of D-gal treatment at various concentrations (0, 5, 10, 20, and 40 g/L). It has been reported by studies conducted over the years that the mRNA and protein expressions of p21 and p16 genes in senescent cells are elevated to sustain the irreversible growth stasis of such cells. Consequently, p21 and p16 are deemed as two crucial regulators for the life cycle of cells [32]. As expected, the results of aging-associated proteins (p21 and p16) illustrated that the processing with D-gal treatment was capable of simulating the aging progress of porcine SCs. Moreover, the occurrence of aging progress also disrupted TJ in SCs which was confirmed by the findings that SCs exposed to 40 g/L D-gal in culture possessed overtly raised protein expressions of Claudin-11, Occludin, and ZO-1. Consistently, a recent study uncovered that the exposure of TM4 cells to 50-150 mmol of D-gal could disrupt TJ function in an aging model [33].

As an antioxidant and anti-inflammatory substance, Curcumin was demonstrated to exert a curing effect on cisplatin [34], aluminum [35], aflatoxin [36], and testicular torsion-induced damage [37]. Moreover, it has been demonstrated that Curcumin can reduce Leydig cell apoptosis and increase testosterone secretion in aged mice [38]. In this research, the role of Curcumin in regulating the aged SCs and its association with the decline in TJ function were examined. The results manifested that in SCs treated with D-gal, the expressions of TJ proteins ZO-1, Occludin, and Cludin-11 were notably reversed by Curcumin, indicating that Curcumin can block aging-induced disruption of TJ function of SCs. Consistently, the latest studies have denoted that Curcumin is capable of repressing and reversing the disruption of TJ function in intestinal Caco-2 BBe cells [39], lung A549 cells [27], and mammary epithelial cells as well [40]. These findings implied that restricting the disruption of TJ function is a vital aspect thanks to that Curcumin has plenty of pharmacological effects.

Once confirming the antiaging-induced TJ function can disrupt the properties of Curcumin, the fundamental molecular mechanism was primarily elucidated. AMPK and SIRT3 are key molecules of aging and related degenerative diseases. In murine ovaries, the activation of AMPK and the expression of SIRT3 are decreased with aging [41]. It was also found *via* the present study that p-AMPK and SIRT3 expressions in young porcine testes were significantly decreased compared with those in old ones, suggesting that the decline of testicular function with aging may be closely related to the decline of AMPK and SIRT3. Moreover, AMPK and SIRT3 have been shown to be essential players in preserving the integrity of blood-brain barrier [42, 43]. It has been demonstrated that Curcumin is able to alleviate liver injury and oxidative stress in the liver tissues obtained from biliary cholestasis mice by means of active AMPK and rising SIRT3 expression [44]. Consistently, decreases in AMPK activation and SIRT3 expression were observed after Curcumin treatment in the present study. Moreover, following the treatment with the inhibitors of AMPK (compound C) and SIRT3 (3-TYP), there was an antagonism effect on Curcumin, and the TJ proteins (namely, ZO-1, Occludin, and Claudin-11) in D-gal-treated SCs presented lowered expressions. Intriguingly, SIRT3 was discovered to activate AMPK in several disease models, according to previous studies [45–47]. Moreover, it has been reported that Lycium barbarum seed oil stimulates testosterone secretion and reduces oxidative stress partly *via* the activation of the SIRT3/AMPK signaling pathway in subacutely aging murine testes, and SIRT3 silently decreases the AMPK phosphorylation in TM4 cells [47]. Nonetheless, there is growing evidence that AMPK has a regulatory effect on SIRT3. It has been demonstrated that high palmitic acid (PA) induces ceramide accumulation to cause the hyperacetylation and dysfunction of mitochondrial protein in oocytes *via* suppressing the AMPK/SIRT3 signaling pathway [48]. Based on another study, the SIRT3-induced AMPK pharmacologic activation represses the oxidative stress while enhancing the mtDNA integrity to limit the development and progression of aging-triggered knee osteoarthritis (OA) in mice [49]. It is also indicated that restraining AMPK using AMPK1*α* siRNA or compound C (AMPK inhibitor) evidently lowers the SIRT3 level, whereas SIRT3 siRNA is unable to remarkably alter the AMPK phosphorylation, implying the role of SIRT3 as downstream target of AMPK. As a result, the aforementioned studies denote that the AMPK and SIRT3 signaling pathways may be overlapped. It was verified by this research that the incubation of cells with D-gal was able to induce aging, as confirmed by the observed increase of AMPK activation and SIRT3 expression after Curcumin treatment. Combined with the treatment of compound C, an opposite effect was obtained, that is, AMPK activation and SIRT3 expression were inhibited. Nevertheless, the treatment combined with 3-TYP could reduce SIRT3 expression, without a remarkable influence on AMPK phosphorylation. The present data suggest that the disruption of Curcumin anti-TJ may be mediated in SCs, at least in part, *via* suppressing the AMPK/SIRT3 signaling pathway.

Oxidative stress is a common pathology seen in many infertile men [50]. It has been shown that antioxidant defense decreases with aging in the male reproductive system [51]. SOD and CAT play pivotal roles in the antioxidant enzymatic defense system [52], whose activities are used as indicators for aging. Being a secondary lipid peroxidation product, MDA is under the stimulation of oxygen free radicals [53]. MDA level is discovered to be elevated in the mitochondria in the case of heart failure [54], and it is extensively regarded as a biomarker of oxidative stress [53]. According to the present study, the old porcine testes presented a higher MDA level, as well as lower SOD and CAT levels compared to the young ones, which is similar to findings of previous studies on murine testes that old testes are under an environment with excessive oxidative stress and enhanced lipid peroxidation. It has been shown that D-gal can induce the senescence of endothelial cells [55] and keratinocytes [56] in a ROS- and mtROS-dependent manner, respectively. As a potential antioxidant, Curcumin has been demonstrated to alleviate the destruction of blood-brain barrier by recovering TJ proteins, inhibit the neuronal apoptosis in lesions mediated by mitochondria, and alleviate cerebral ischemia-reperfusion (I/R) injury through downregulating ROS accumulation in a mouse model of transient cerebral ischemia [57]. In the present study, it was revealed that the levels of total ROS and mtROS in SCs were markedly increased by D-gal, while such an effect was abolished by Curcumin, and the antioxidant performance of Curcumin relies on the activation of the AMPK/SIRT3 signaling. There is ever-increasing evidence that SOD2 is located in the downstream of SIRT3 in mitochondria while attenuating mtROS [18]. As an iron/manganese superoxide dismutase, SOD2 is a crucial player in modulating the superoxide by-products of oxidative phosphorylation [19]. In this study, the expression level of SOD2 protein was significantly decreased in old porcine testes compared to that in the young ones, and *in vitro* results demonstrated that compound C and 3-TYP could abolish the stimulating effect of Curcumin on SOD2 expression in D-gal-treated SCs. Moreover, after mito-TEMPO was eliminated by mtROS, the degrading TJ protein expressions (e.g., ZO-1, Occludin, and Cluadin-11) triggered by D-gal were significantly inhibited. Generally, valuable complementary findings were acquired in this study that the AMPK/SIRT3/SOD2 signaling pathway implicated inhibits the generation of ROS and mtROS in the case of age-related TJ function disorder in SCs.

It has been recognized that ROS and mtROS are key triggers for the formation of NLRP3 inflammasome [17]. The signaling of NLRP3 inflammasome initiates the generation of IL-1*β* and other proinflammatory mediators and then promotes the release of proinflammatory cytokines like TNF-*α*, causing systemic chronic inflammation in the case of aging [10]. It was uncovered through this study that NLRP3 and IL-1*β* expressions exhibited obvious increases in old porcine testes in contrast to those in young ones, suggesting that the old testis is under an inflammation environment. Moreover, our *in vitro* results showed that Curcumin and mito-TEMPO ameliorated D-gal-induced NLRP3 inflammasome activation and IL-1*β* release, indicating the potential effect of Curcumin through activating the AMPK/SIRT3/SOD2/mtROS signaling pathway in SCs. IL-1*β*, an important mediator of inflammation and tissue damage, can accumulate augmenting inflammation and aggravate blood-brain barrier disruption by disordering TJ function [58]. IL-1*β* performs its functions in an autocrine/paracrine manner by virtue of type I IL-1 receptor (IL-1R1). The conjugation of IL-1R1 with IL-1Ra, a natural cytokine responsible for preventing the biological response to IL-1, can suppress the IL-1R1 activation [59]. The expression levels of TJ proteins were examined in SCs to elucidate the mechanism of aging's disrupting BTB integrity in aged mice testes through activation NLRP3 and IL-1*β* production. Our results show that MCC950 (the inhibitor of NLRP3) and IL-1Ra rescue the expression of ZO-1, Occludin, and Claudin-11 in D-gal-induced SCs. These data strongly suggested that the suppression of NLRP3 activation and IL-1*β* release was involved in Curcumin's rescuing senescent SCs' tight junction. Based on the *in vitro* data, Curcumin could serve as a feasible choice for treating D-gal-associated TJ dysfunction in SCs by inactivating NLRP3 inflammasome, which is partially attributable to the fact that the AMPK/SIRT3/SOD2 signaling pathway is activated to inhibit the production of ROS plus mtROS.

Furthermore, whether Curcumin significantly affects the spermatogenic function of mice suffering from D-gal-induced aging was explored as well, which is potentially conducive to testifying the effects on rescuing the TJ function in SCs to recover spermatogenesis. Our data showed that Curcumin treatment improved the testis histology and sperm motility, but reduced the deformity rate in mice with D-gal-induced aging, suggesting that the testicular spermatogenic function is restored in aging mice. Besides, the expression of ZO-1, Claudin-11, and Occludin presented increase in D-gal-induced aging testes following Curcumin treatment, suggesting that Curcumin can restore spermatogenic function in aged mice by restoring BTB function. A recent study has shown that ROS and inflammatory response are crucial players in disrupting BTB integrity [5]. In our study, in comparison with those in the young mice, MDA level and NLRP3 and IL-1*β* expressions were significantly elevated, while the SOD and CAT levels were lowered in the testicular tissues of the aging mice, suggesting that the testes are under a microenvironment with oxidative stress and inflammation. However, Curcumin treatment increased the content of SOD and CAT and decreased MDA content together with NLRP3 and IL-1*β* expressions, suggesting that the antioxidant and anti-inflammatory effects of Curcumin are the key factors in restoring BTB integrity in aging testes. Moreover, consistent results of examinations of the AMPK/SIRT3/SOD2 signaling pathway and NLRP3 inflammasome in the testes are also obtained from the *in vitro* experiments, assuring the molecular mechanisms of Curcumin in recovering the TJ function in SCs. The data from this study could corroborate that BTB disruption is present in the testes during aging and emphasize that activating the AMPK/SIRT3/SOD2 signaling pathway to prevent the ROS from triggering NLRP3/IL-1*β* is an underlying mechanism of Curcumin in improving TJ function in SCs to recover the aging spermatogenic function of the testis.

In conclusion, Curcumin was proven by the existing data to be able to recover the TJ function in senescent porcine SCs while increasing the spermatogenic ability in the mouse model of aging. These effects were related to the activation with the AMPK/SIRT3/SOD2/mtROS signaling pathway for repressing NLRP3 inflammasome in SCs. The overall findings of this study explore a new route for researching the mechanism of Curcumin-triggered protective function in age-associated male reproductive disorder, revealing that inhibiting NLRP3 inflammasome by targeting the AMPK/SIRT3/SOD2/mtROS signaling pathway may serve as an effective therapeutic method to treat age-related BTB disorders.

## Data Availability

The data will be made available after being requested from the corresponding author.
